# Antimicrobial Peptides (AMPs) and the Microbiome in Preterm Infants: Consequences and Opportunities for Future Therapeutics

**DOI:** 10.3390/ijms25126684

**Published:** 2024-06-18

**Authors:** Janina Marissen, Lilith Reichert, Christoph Härtel, Mats Ingmar Fortmann, Kirstin Faust, Delfina Msanga, Jürgen Harder, Michael Zemlin, Mercedes Gomez de Agüero, Katja Masjosthusmann, Alexander Humberg

**Affiliations:** 1 Department of Pediatrics, University Hospital Würzburg, 97080 Würzburg, Germany; marissen_j@ukw.de (J.M.); reichert_l@ukw.de (L.R.); 2 Würzburg Institute of Systems Immunology, Max-Planck Research Group, University of Würzburg, 97078 Würzburg, Germany; mercedes.gomez@uni-wuerzburg.de; 3 German Center for Infection Research, Site Hamburg-Lübeck-Borstel-Riems, 23538 Lübeck, Germany; 4 Department of Pediatrics, University Hospital Schleswig-Holstein, 23538 Lübeck, Germany; matsingmar.fortmann@uksh.de (M.I.F.); kirstin.faust@uksh.de (K.F.); 5 Department of Pediatrics, Bugando Hospital, Catholic University of Health and Allied Sciences, Mwanza 33109, Tanzania; deromsah@gmail.com; 6 Department of Dermatology, Venerology and Allergology, Quincke Research Center, Kiel University, 24105 Kiel, Germany; jharder@dermatology.uni-kiel.de; 7 Department of General Pediatrics and Neonatology, Saarland University Medical Center, 66421 Homburg, Germany; michael.zemlin@uks.eu; 8 Department of General Pediatrics, University Children’s Hospital Münster, 48149 Münster, Germany; katja.masjosthusmann@ukmuenster.de (K.M.); alexander.humberg@ukmuenster.de (A.H.)

**Keywords:** antimicrobial peptides, microbiome, premature infants, dysbiosis, epidermis, sustained inflammation, innate immune system, microbiota-regulation peptides/proteins

## Abstract

Antimicrobial peptides (AMPs) are crucial components of the innate immune system in various organisms, including humans. Beyond their direct antimicrobial effects, AMPs play essential roles in various physiological processes. They induce angiogenesis, promote wound healing, modulate immune responses, and serve as chemoattractants for immune cells. AMPs regulate the microbiome and combat microbial infections on the skin, lungs, and gastrointestinal tract. Produced in response to microbial signals, AMPs help maintain a balanced microbial community and provide a first line of defense against infection. In preterm infants, alterations in microbiome composition have been linked to various health outcomes, including sepsis, necrotizing enterocolitis, atopic dermatitis, and respiratory infections. Dysbiosis, or an imbalance in the microbiome, can alter AMP profiles and potentially lead to inflammation-mediated diseases such as chronic lung disease and obesity. In the following review, we summarize what is known about the vital role of AMPs as multifunctional peptides in protecting newborn infants against infections and modulating the microbiome and immune response. Understanding their roles in preterm infants and high-risk populations offers the potential for innovative approaches to disease prevention and treatment.

## 1. Introduction

### The Preterm Infant and Its Risk for Immune Mediated Injuries

According to the World Health Organization (WHO), premature birth is defined as a live birth before the 37th week of pregnancy or before the 259th day after the last menstruation [1]. Various factors are known to contribute to preterm birth. Inflammatory processes, potentially leading to the rupture of membranes, preterm labor and chorioamnionitis, play a crucial role [2,3]. Additional causes include pregnancy-related conditions such as pre-eclampsia, maternal or fetal indications, and spontaneous preterm labor [4,5]. Premature babies are more susceptible to inflammation-related complications, which are mainly observed in the context of antenatal exposure to inflammation, perinatal asphyxia, or acute and chronic postnatal inflammatory processes. These processes can induce developmental disturbances of the immature organs and lead to long-term impairments and health restrictions [2], primarily in neurological (including cognitive, neurological, motor, and sensory limitations) and pulmonary development.

One important inflammatory disease of the preterm infant with devastating consequences for long-term neurological sequelae is an inflammatory alteration of the gut, named necrotizing enterocolitis (NEC). NEC can be rapidly progressive and increases the mortality and morbidity in preterm infants [6]. The pathophysiology of NEC is not yet fully understood but is thought to be multifactorial, involving the interaction of dysbiosis, immaturity of the intestinal wall, and the immune system of the vulnerable gastrointestinal tract [7,8,9]. An excessive inflammatory response with consecutive damage to the microglia is a possible cause of neurocognitive deficits observed after NEC [6,10]. White matter abnormalities are strongly correlated with reduced intelligence quotient scores and poorer motor outcomes later in life [11,12,13].

Exposure to an inflammatory environment also contributes to lung impairment [14,15,16,17,18]. In the postnatal course, up to 50% of premature babies develop a phenotype of chronic lung disease, i.e., bronchopulmonary dysplasia (BPD) [19]. The histological characteristics of BPD include changes in lung structure with decreased septation, vascularization, number of alveoli, and simplified alveolar structures. This results in a reduced ability to exchange gases, which predisposes to chronic obstructive pulmonary disease (COPD) in early childhood [19]. As such, premature babies show significantly reduced forced expiratory volume in one second [20] and increased airway resistance [21,22] leading to reduced exercise capacity in daily life [23]. Important triggers of BPD include mechanical shear injuries from positive pressure ventilation, the use of supplemental oxygen, and the onset of inflammatory processes, which may begin prenatally and all cause lung inflammation [19].

Preterm infants born from an inflammatory environment, particularly with a maternal diagnosis of chorioamnionitis, present with increased lung inflammatory markers and are at a heightened risk to develop BPD [24]. In animal models, the application of lipopolysaccharide (LPS) or Escherichia coli endotoxin as stimuli for a pro-inflammatory response has been shown to be detrimental to alveolar and vascular development [18,25,26,27]. Infants with a history of infection have increased incidences of BPD [28]. However, there is inconsistency in the scientific literature regarding the association between histological or clinical chorioamnionitis and BPD, as their underlying mechanisms and the role of prenatal inflammation remain not fully clarified.

At birth, the immune system is responsible for tolerizing colonization to the host’s benefit and priming to fight potential infections. A complex process involving molecular, cellular and epigenetic programs helps modulate the immune system to allow microbial colonization while avoiding exuberant inflammation and autoimmunity. While the cells of the acquired immune system need time to mature and experience antigenic exposure, the innate immune system is present at birth and does not require such arrangements. Key components of the innate immune system include tight junction complexes, epithelial cell layer integrity, cells such as phagocytes, natural killer cells, and antigen-presenting cells, and humoral factors such as cytokines, antimicrobial peptides (AMPs), and complement factors [29,30,31]. These components are present at epithelial and endothelial barriers and in tissue fluids, providing a rapid and broad protective shield against various pathogens.

The immature characteristics of the premature immune defense system explain the susceptibility of preterm infants to diseases of infectious and inflammatory etiology, which can lead to long-term complications. Additionally, preterm infants are at an increased risk of abnormal colonization of the gut, disrupting bacterial homeostasis, a condition referred to as dysbiosis. This dysbiosis is thought to lead to further inflammation, possibly causing irreversible damage to organs, including the lungs, the brain, and intestines [6,32,33].

## 2. Purpose of the Review

As preterm infants are at increased risk for immune-mediated injuries, a thorough understanding of the mechanisms and interactions of the immune system in this population is essential and might help us to improve outcomes in this vulnerable cohort. In the following review, we will discuss the role of AMPs as factors of the innate immune system in preterm infants and their role in various aspects of preterm health. The focus will be on the interactions of AMPs with the microbiome at different sites and their possible roles in health and disease.

## 3. Antimicrobial Peptides

AMPs form an important part of the innate barrier [34] and are produced by prokaryotic and eukaryotic organisms, including microorganisms, plants, insects, vertebrates, and mammalians [34,35,36,37,38]. In humans, AMPs are ubiquitously found in immune cells such as neutrophils, monocytes, and macrophages, and are also released from epithelial cells of the skin and mucosal surfaces, and are present in body fluids [39,40,41,42,43,44,45]. They can therefore act on the skin, respiratory tract, gastrointestinal tract, urinary tract, and are also found in breast milk. Human host AMPs show different expression patterns with age [46]. Due to their additional immunomodulatory activities, AMPs are also called “host defense peptides ”. Recently, the term “microbiota-regulating peptides/proteins” has been proposed, as AMP act on both the regular microbiota and invaders [47].

Since their first description by Alexander Fleming, who had discovered lysozyme [48], more than 3000 AMPs [49] have been identified, especially in the last two decades. AMPs typically consist of 5–50 amino acids, most of which are positively charged, hydrophobic and amphipathic in structure [50,51,52,53]. Due to their positive charge, AMPs can bind to lipopolysaccharide and lipoteichoic acid, which are key components of the bacterial cell wall [54,55,56,57,58,59]. Based on their composition, size, and structure, AMPs can be classified into alpha helix peptides, beta sheet peptides, or loop peptides, although more complex structures also exist [60]. AMPs often exhibit broad-spectrum antimicrobial activity by directly or indirectly killing microbes [61]. They can directly kill microbes by forming pores in the bacterial membrane [55,62,63], inhibit molecular functions such as bacterial nucleic acid synthesis, protein synthesis, and cell wall synthesis by entering bacterial cells [64], or direct cytokines and modulate inflammatory reactions to the site of infection [65]. They also induce angiogenesis, promote wound healing, inhibit pro-inflammatory reactions, modulate adaptive cellular immune responses, and act as chemoattractants for immune cells [34,35,36,37,50,51,52,53]. Therefore, AMPs play a role in controlling infections [66] and may have therapeutic potential even in mixed infections or biofilm-associated infections [67]. However, bacteriocins (AMPs derived from bacteria), often have a limited spectrum of antimicrobial activity against other bacteria that are phylogenetically related [68].

For research purposes, AMPs may be collected, according to the body site, through cutaneous lavage probes, throat swabs/nose brushing or directly from blood, urine, and breast milk. Enzyme-linked immunosorbent assays (ELISA) are commonly used for quantification [69,70]. Studies in neonates generally reveal decreased concentrations of circulating, intracellular, and epithelial AMPs in preterm infants, which may contribute to reduced immune protection [70,71,72,73,74]. However, we could recently demonstrate that AMP concentrations on the skin do not differ between preterm and term-born infants and are not gestational age dependent [69]. Moreover, increased levels of AMPs were noted in infants born to mothers with a history of chorioamnionitis, which might act as a confounder or modifier when assessing the effect of gestational age on AMP levels [69]. Studies not adjusting for the cause of preterm delivery should be interpreted with caution [75,76].

The complex dynamics of AMP levels in developmental organisms, particularly in early-life commensal colonization and protection against infection, are currently under scientific investigation. However, many gaps in knowledge on the microbe-immune processes exist but provide an interesting target for preventing distortions in microbiome composition (dysbiosis) and its consequences.

## 4. The Microbiome

The human microbiome plays a central role in various aspects of infant and later-life physiology and health. It is widely accepted that the establishment of the microbiome begins during and immediately after birth, although some studies have detected traces of microbial DNA in the placenta and amniotic fluid [77,78]. Nevertheless, it is important to acknowledge that highly sensitive molecular techniques may have contamination issues [79]. Nonetheless, it is well accepted that the maternal microbiome, its metabolites and maternal inflammation collectively exert significant impact on the developing fetus during pregnancy [2] and may influence the gene expression encoding for AMPs [80]. However, the interplay of microbiome, metabolome, and AMPs is not well understood yet and data are scarce.

At the time of birth, the microbiome across various body sites is relatively homogenous [81]. Subsequently, the infant’s microbiome undergoes rapid body-site specific diversification and maturation in the days, months, and years following birth. Recent research indicates that approximately 58% of the microbiome in various body sites of full-term infants originates from maternal sources [82]. Factors influencing the microbiome composition include gestational age, delivery mode, environmental factors (particularly within a hospital setting), infant nutrition (breast milk or formula), antibiotic treatments and the body sites themselves, each with different niche factors [81,83]. While the initial colonization is dependent on the mode of delivery, research has shown that alternative sources of microbial transmission can occur across various niches and body sites. For instance, infants born via Caesarean section (CS) can benefit from the microbiome present in breast milk and frequent bonding [82]. Nevertheless, the aforementioned factors may interfere with the development of a healthy microbiome, with changes in the metabolome or in AMP patterns.

Despite extensive studies on the gut microbiome of infants and its association with diseases such as sepsis or NEC [84,85], other microbiome niches in infants with lower microbial biomass, such as skin or airway microbiome, remain less characterized. Nevertheless, microbiome patterns in these body sites may play a role in disease development [83,86,87]. Particularly in preterm infants, who are highly vulnerable to infectious and inflammatory diseases and subsequent sequelae, there is still a lack of longitudinal data with the microbiome as target for prevention.

Regarding the skin microbiome in infants, there is a limited number of studies available [88]. The initial skin microbiome is primarily influenced by the mode of delivery [81,83]. At birth, term infants born via CS tend to exhibit a microbiome resembling that of the maternal skin, enriched in *Staphylococcus*, *Corynebacterium*, *Streptococcus*, and *Proprionibacterium* species (spp.) In contrast, vaginally born infants tend to have a microbiome dominated by *Lactobacillus* spp. of vaginal origin. However, these differences tend to diminish by 6 weeks of age [81], although some studies still report disparities at 6 months of age [83]. The skin microbiome of preterm infants is characterized by a predominance of Firmicutes, particularly *Staphylococcus* spp., known for its crucial role in immune tolerance and function. As a typical skin commensal, *Staphylococcus epidermidis* (*S. epidermidis)* colonizes the skin immediately after birth and dominates the skin microflora in the first months of life [89]. Further colonizers include Actinobacteria (e.g., *Corynebacterium* spp.), Gammaproteobacteria (e.g., *Escherichia coli*, *Enterobacter* spp.), Bacilli (e.g., *Streptococcus* spp.) and Bacteroidetes (e.g., *Prevotella* spp.). In contrast, the skin microbiome of term infants exhibits a higher abundance of Proteobacteria and Bacteroidetes [83,90]. *Staphylococcus* abundance tends to decrease with age [83]. Overall, the skin microbiome of infants is strongly influenced by the maternal microbiome and exhibits the highest similarity to the maternal and adult microbiome [81,91]. Nevertheless, significant temporal variations in the microbial composition occur during the first year of life with increasing diversity [83].

Another low-biomass microbiome site is the lower airway microbiome, which is challenging to access for research purposes. In the lower respiratory tract, one study identified three distinct microbial profiles detectable as early as 24 h after birth. These profiles displayed varying age-dependent potentials for mucosal defense mechanisms. The profiles were dominated by either *Staphylococcus* spp., *Ureaplasma* spp., or a mixed composition of *Streptococcus*, *Neisseria*, *Prevotella*, *Porphyromonas*, *Veillonella and Fusobacterium* genera. Gestational age emerged as a major influencing factor, with all preterm infants predominantly exhibiting either the *Staphylococcus* or *Ureaplasma* profile. Delivery mode appeared to have an impact solely on preterm infants. Within two months, the airway microbiome tended to mature and stabilize in terms of diversity [92]. Other studies have confirmed the dominance of *Staphylococcus* spp., *Ureaplasma* and Proteobacteria in preterm infants [93,94]. A healthy lung microbiome composition typically contains phyla such as Bacteroidetes, Actinobacteria, and Firmicutes, whereas an abundance of Proteobacteria (e.g., *Haemophilus*, *Moraxella*) is associated with viral infections [95].

In the gut, infants born via CS tend to lack certain bacteria that would have otherwise been transferred during a vaginal birth, such as *Bifidobacterium* spp. Other genera, notably *Bacteroides* spp. tend to diminish during the first two weeks of life [82].

In the meconium of preterm infants, we have observed a predominance of *Bifidobacterium*, *Staphylococcus* and *Enterococcus* spp. Notably, *Bifidobacteria* exhibited a negative correlation with pathogenic bacteria [96]. Another study identified a relatively high abundance of *Escherichia* und *Klebsiella* in term infants’ stool [81]. The maturation of the gut microbiome into an adult-like composition occurs during the first year of life and is contingent upon factors such as nutrition and the duration of breastfeeding [97], but also on antibiotic treatments. With increasing postnatal age, preterm infants’ gut microbiome matures from a *Staphylococcus* and *Enterococcus*-dominated composition to *Enterobacter* and *Bifidobacteria* domination [98]. A randomized controlled study in preterm infants showed that administration of a probiotic mixture of *Bifidobacteria* could accelerate the transition into a mature gut microbiome with a favorable metabolic and immune milieu [99]. AMPs in the gut may support this by controlling the bacterial load of specific gram-negative and even antibiotic-resistant bacteria [100].

### Consequences of an Altered Microbiome in Preterm and Term Health

As mentioned above, the skin microbiome of preterm infants is characterized by lower diversity, possibly due to gestational age at birth and dominance of staphylococci, especially *S. epidermidis*. However, this dominance may also be associated with late-onset sepsis (LOS) [90,101]. While our understanding of the development and role of the gut microbiome in preterm and newborn infants has expanded significantly over the past decades, the development of the skin microbiome and its consequences are less well understood.

An infant’s nasopharyngeal microbiome dominated by *Staphylococcus* [97] and shifts towards pathogenic bacteria in the nasopharyngeal microbiome (e.g., *Moraxella*) have been observed to precede respiratory infections, wheezing, and allergic sensitization in later childhood. Through the gut–lung axis, dysbiosis in gut microbiota may contribute to food allergies and asthma [95]. In preterm infants, the airway microbiome has been associated with BPD, characterized by reduced diversity and abundance of Firmicutes and *Lactobacilli*, alongside an increased abundance of Proteobacteria, *Ureaplasma*, *Acinetobacter*, *Staphylococcus* and *Klebsiella* spp. in tracheal aspirates [102].

Gut dysbiosis, characterized by decreased abundance of *Bifidobacteria* and increased abundance of Gammaproteobacteria, has been observed to precede neonatal sepsis and NEC in preterm infants [84,85,103]. Similarly, increased abundance of Bacilli, specifically coagulase-negative *staphylococci*, in the gut has been shown to precede infection in preterm infants [104]. In this cohort, lower abundances of Gammaproteobacteria were found in infants who developed culture positive LOS [105], suggesting that certain phyla might be protective at a certain time point while associated with higher risk for inflammatory diseases at other time points. An *Enterococcus*-enriched microbial pattern has been associated with impaired outcomes at two years of age [106]. Furthermore, deviations in the skin microbiome, along with gut dysbiosis, have been linked to later development of atopic dermatitis and allergic disease [83]. Host–microbiome interactions play a role in all these associations. In the following parts, we will discuss the possible interactions of antimicrobial peptides with the host and microbiota.

## 5. Antimicrobial Peptides and Interactions with the Host

Defining the microbiome as the result of an interaction between the microbiota and host habitat [107] integrates the important role of an existing host control over the microbial environment [108,109,110]. The production of pathogenic organisms can be prevented by competitive exclusion, production of specific antimicrobial/microbiota-regulating factors, or stimulation of cells to secrete antimicrobial factors. AMPs play an important role in maintaining a uniform colonization by microorganisms of the surfaces, especially of the skin, lungs and intestines, and are therefore an important player of host-derived control factors.

For example, as a typical skin commensal, *S. epidermidis* produces antimicrobial substances and toxins (for example, phenol-soluble modules) [104], which repel other pathogens like *S. aureus*, thus stabilizing the skin microflora. The observation that a large number of *S. epidermidis* microbes can be found in the first few months of life and then decrease over time suggests that *S. epidermidis* plays a central role in the initial phase of early immune response and defense against pathogens [89,111,112]. However, an important discovery of the last few decades is that *S. epidermidis* is much more than a single microbe. Individual strains differ dramatically in their genome, pro-inflammatory potential, biofilm formation, and interaction with the host’s immune system [113,114,115]. Some strains of *S. epidermidis* are common causative agents of LOS in preterm infants and neonates [11,116,117] and are associated with inflammatory diseases in preterm infants such as BPD [110]. Integrated defense mechanisms against potential competitors appear to benefit *S. epidermidis* under increased selection pressure [118]. This nosocomial selection pressure, with a shift towards a pathogenic profile, suggest an important component to be influenced in reducing inflammatory disease in preterm infants [105,119] and antibiotic use [101]. In the following part we will discuss AMPs in different organs. A summary of AMPs and possible interactions with the microbiome in the respective body departments is given in Table 1.

### 5.1. Antimicrobial Peptides on the Skin

The skin plays a special role as an immune barrier and protection against infections. AMPs on the skin surface form an important part of the skin’s innate immune barrier. Defensins (human beta-defensin [hBD]), cathelicidins (LL-37), psoriasin, RNase 7, dermcidin, and adrenomedullin have been identified in human skin [120,121,122,123,124,163]. Due to their broad antimicrobial properties against bacteria, viruses and fungi, these peptides provide effective immune protection. For instance, RNase 7 can prevent *S. aureus* colonization in skin explants and exhibit high in vitro antimicrobial activity against many Gram-positive and Gram-negative bacteria and yeast [122,164,165,166]. Additionally, AMPs serve as important effector molecules in the regulation and interaction of the innate and adaptive immune systems, controlling cell migration, proliferation and differentiation, as well as modulating Toll-like receptors (TLR) and cytokine production [125,126,127]. Stimuli such as the expression of pro-inflammatory cytokines, mechanical injury, or inflammation lead to keratinocyte activation and upregulation of AMPs in the skin [167]. Individual AMPs exhibit distinct properties: isolated hBD-1 demonstrates only weak antimicrobial activity, but when combined with other defensins, it exhibits significantly stronger antimicrobial properties. hBD-2 is less active against *S. aureus*, whereas hBD-3 is highly bactericidal against *S. aureus* [168].

The expression of AMPs on the skin is modulated by *S. epidermidis*. By stimulating TLR-2, *S. epidermidis* induce the expression of hBD-2 and hBD-3 in human keratinocytes [128]. Given these abilities of specific microorganisms to induce particular AMPs, there is reason to believe that skin dysbiosis also leads to an altered AMP profile.

Studies in amphibians have shown that the growth of pathogenic germs on the skin of these animals is synergistically inhibited by the interaction of commensals and AMPs in vitro [129] and AMPs promote the stabilization of the cutaneous microbiome [130,169].

AMPs serve as key regulators of the microbiome and immune modulators of defense against pathogens. Our group recently demonstrated that psoriasin and RNase 7 level in the skin accelerate expression over time and that levels do not differ between preterm and term infants with respect to day of life [69].

### 5.2. Antimicrobial Peptides in the Lungs

The lung epithelium is rich in immune cells including alveolar macrophages and epithelial cells, which are all able to produce AMPs to protect the lungs from pathogens entering the airways [43,170,171,172,173,174]. Most data on preterm and term infants derive from in vitro experiments, studies of fetal lungs and the bronchoalveolar fluid of ventilated neonates. These studies have documented the presence of AMPs in developing human lungs, including the expression of α- and β-defensins, LL-37, elafin, and secretory leukocyte protease inhibitors (SLPI) [70,132,133,134]. However, due to ethical limitations, data on the role of AMPs in the neonatal lung are scarce and still require further evaluation. Both cathelicidins and defensins demonstrate important immunomodulatory functions in the lungs. Whether AMP levels are lower in preterm infants compared to term infants remains to be established [70,175,176], but hBD-2 seems to be the predominant defensin in the neonatal lung [70] and is induced in pulmonary epithelial cells in response to LPS through the activation of nuclear factor kappa B (NF-κB) [177,178]. In neonatal tracheal aspirates hBD-2 increased with gestational age, whereas hBD-1 was barely found [70]. The reduced hBD-2 concentration could allow specific bacteria the colonization and infection of the respiratory tract of preterm infants more easily. For example, *H. influenzae*, a common commensal in the human respiratory tract, may cause diseases like BPD in the neonatal lung due to reduced hBD-2 levels.

In chorioamnionitis, AMPs play a crucial role in regulating the immune response. Early in utero suppression through chorioamnionitis might be important for the precise regulation of the fetal inflammatory response and tissue reorganization in the preterm lung. Specifically, in pulmonary cells, the human cathelicidin LL-37 and defensins enhance epithelial cell proliferation and induce signaling pathways to activate airway epithelial cells [135,136,175,176,177,178]. Fetal sheep exposed to intra-amniotic LPS prior to preterm delivery exhibited decreased concentrations of cathelicidins and defensins one day after intra-amniotic exposure to LPS, but cathelicidins increased eight days after LPS exposure [179]. This suggests their potential role in tissue repair following injury, as cathelicidins enhance epithelial cell proliferation and accelerate wound closure [135,136]. This observation might explain why antenatal inflammation is inconsistently associated with chronic lung disease, depending on the time of exposure [180,181]. Interestingly, after dexamethasone administration, hBD-2 mRNA expression can be downregulated by dexamethasone whereas hBD-1 synthesis is induced [70]. This is of particular interest, as use of postnatal corticosteroids could play a role in preventing and treating BPD [182].

The antiproteases elafin and SLPI are constitutively produced by neonatal lung immune cells of neonates [137] and may help prevent ventilator-induced lung injury [138,139]. In mechanically ventilated preterm neonates with respiratory distress syndrome, lower SLPI concentrations have been reported [140] and associated with the development of ventilator-induced lung injury [141].

These few observations highlight the need for further research to better understand the role of AMPs in preterm infants and their responses to developmental challenges such as antenatal inflammation, postnatal ventilation and infections.

Preterm infants are at increased risk for long-term sequelae associated with viral infections [183,184]. Therefore, protective strategies are urgently needed to reduce the long-term complications of viral infections during the neonatal period. While vaccines are considered the best prophylactic measure against various viruses (e.g., respiratory syncytial virus (RSV), influenza), most viral pathogens are not covered by vaccines. Due to their broad-spectrum activity, AMPs could provide ideal protection against different viral strains. Some studies have shown in vitro antiviral effects for defensins and cathelicidin against Herpes simplex virus, RSV [185], and influenza [186]. However, in vivo studies are very limited and suggest that the inhibition of virus replication by AMPs might not be relevant in vivo [187]. Nonetheless, novel engineered AMPs derived from natural protein modifications could serve as potential antiviral agents leading to novel antiviral therapeutics [188,189].

### 5.3. Antimicrobial Peptides in the Gastrointestinal Tract

The maintenance of a homeostatic gut microbiome and protection against dysbiosis during the vulnerable newborn period seems essential, as particularly gut dysbiosis can lead to severe inflammatory, metabolic, neurologic, cardiovascular, and gastrointestinal disease in preterm infants [2,142,143].

The host’s immune system and intestinal epithelium interact at the level of a microbiome-stimulated immune response, which must be continuously controlled via a low-grade stimulated immune system [190]. Research conducted on germ-free mice has shown that specific intestinal AMPs are produced independently of signals from the microbiota [191], while others are released into the gut in response to stimulation from molecules associated with microbes and inflammatory cytokines [192,193].

At this point, AMPs play a major role as molecular regulators. Most notably, α-defensins, which are secreted by intestinal Paneth cells (PC) [144], are able to maintain a balanced microbiome. It has been shown that a reduced functionality of PCs is associated with a decrease in the expression of α-defensins and has been linked with overgrowth of adherent-invasive *Escherichia coli* (family Enterobacteriaceae) [147] and inflammatory bowel diseases [144,145,146]. Mice with deletion of PC and drop in the expression of α-defensins develop intestinal dysbiosis with increased activity of inflammatory pathways in the ileum [148].

Some preterm infants develop intestinal inflammation leading to damage of the intestinal epithelial barrier, known as NEC, which can be potentially fatal [194]. An important research focus in the field of NEC pathogenesis is to reveal the mechanisms of intestinal injury, with apoptosis and abnormal autophagy emerging as important contributors to barrier disruption and the development of NEC [194]. In normal physiology, autophagy participates in the repair process of the intestinal barrier and helps to maintain its integrity. Against this background, the observation that patients with severe NEC have a reduced concentration of beta defensins seems to be relevant [71]. It could be shown that treatment with hBD3 in the neonatal rat model resulted in intestinal epithelial cell migration and a reduction in the severity and mortality of NEC [195] via a down-regulation of excessive autophagy through hBD3-mediated protection [196]. This might give a therapeutic potential of AMPs in the prophylaxis and treatment of this fatal gastrointestinal disease.

The use of breast milk in preterm infants is an important factor in reducing the incidences of NEC [197]. Breast milk contains a large number of bioactive peptides that exhibit multiple functions, including anti-inflammatory, immunoregulatory, and antimicrobial activities. It has been shown that breast milk microbiome differs between mothers of preterm and term infants [198]. Hormones and cytokines also vary based on gestational age, influencing the anti-inflammatory properties of breast milk. Studies have observed that immune factors in preterm milk may increase due to compensatory mechanisms during preterm labor or maternal systemic inflammation, but these factors tend to decrease over time, making term and preterm milk more alike as the baby grows older [149,199].

Notably, breast milk contains various AMPs, such as lactoferrin (LF), lysozyme, LL-37, α- and β-defensins [149]. Among these, LF is particularly abundant and possesses the ability to effectively inhibit bacterial growth [150], as well as prevent the epithelial attachment of many bacterial pathogens via multiple modes of action [200].

Due to its diverse properties, LF supplementation has been studied as a potential treatment for bloodstream infections and NEC in very low birth weight infants. Recent randomized controlled trials involving infants have suggested that LF supplementation of children’s feeds could reduce the risk of neonatal sepsis [151] and decrease the duration of diarrheal illness [152].

Various proteins in human milk, such as mucin MUC1, MUC4, and β-casein, contribute to preventing the adhesion of enteric pathogens to the intestinal lumen, promoting pathogen clearance, and protecting against antimicrobial resistant pathogens. There is significant interest in developing supplements containing human milk that could support or strengthen the natural defense mechanisms against infections or intestinal injury [201]. Further research is needed to determine if these compounds are safe and effective to protect children from colonization or infection with antibiotic-resistant bacteria.

### 5.4. Antimicrobial Peptides in the Blood

AMPs are consistently present in plasma, serving as a continuous general defense mechanism against potential invading pathogens. Some of these AMPs, such as defensins, LL-37, and bactericidal permeability-increasing protein (BPI), can be produced and released by cells through TLR activation by microbial signals.

In cases of bacterial bloodstream infections, the concentration of BPI in plasma tends to be higher compared to healthy infants [73,159,160,161,162]. Additionally, newborns born to mothers who experienced amniotic infections have higher levels of certain AMPs (LF, BPI, HNP-1, HNP-2, and HNP-3) in their cord blood [202].

However, neonates generally have lower intracellular levels of AMPs compared to adults. This includes lower levels of LL-37 and BPI in neonatal blood and neutrophils. Reduced BPI in neonatal neutrophils is associated with a diminished ability to kill bacteria. It remains uncertain whether the levels of AMPs within an infant’s cells or plasma impact their risk of developing a bloodstream infection or their clinical outcomes following such an infection. Measuring AMP levels in blood or cells has limitations in understanding these differences between neonates and adults. Instead, it may be more pertinent to investigate functional deficiencies in the innate immune response of neonates, such as impaired bacterial killing due to defective neutrophil extracellular traps formation in vitro [203,204].

## 6. Clinical Implications, Future Perspectives and Research Hypotheses/Gaps

**Figure 1:**
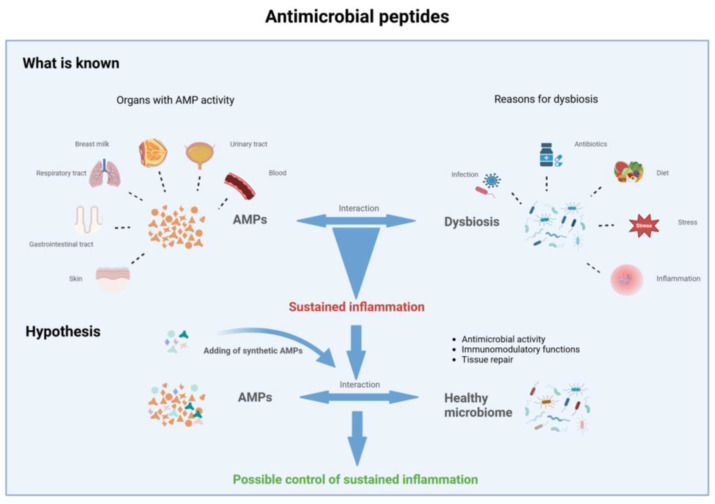
Antimicrobial peptides and sustained inflammation.

AMPs hold promise as potential therapeutics or adjunctive agents to reduce the duration of antibiotic treatment and mitigate inflammation caused by microbes and their products (Figure 1).

AMPs have potential in preventing and treating bacterial infections in infants, particularly in high-risk groups like premature and low birth weight infants, as well as in mixed or biofilm-associated infections [38,67]. Studies have shown that AMP supplementation, either alone or in combination with probiotics, can reduce the incidence of LOS, invasive fungal infections, NEC and lung injury in very low birth weight (VLBW) infants by altering the intestinal microbiota [16,152,155,156,157,158]. However, the use of peptide-based immunotherapies is still in its early stages, but results from several animal models hold promise for future investigations. For example, the use of synthetic AMPs as adjunctive therapeutics in in vivo models could demonstrate reduced inflammatory response. After infection with *S. aureus*, additional administration of AMPs led to a reduced inflammatory response by downregulating proinflammatory cytokines [205]. It is likely that AMPs can control early inflammation in severe infections and attenuate excessively damaging regulatory circuits. Protective effects after AMP injection have demonstrated reduced inflammatory derived brain lesions through LPS induction [206] or even after plasmodium-induced cerebral malaria [205]. Other synthetic AMPs show potent efficacy in anti-biofilm activity, especially when administered with antibiotics [207,208].

Infants, especially preterm or VLBW infants, typically have relatively low levels of circulating and intracellular AMPs, rendering them more susceptible to infections. Research should investigate whether supplementing these infants with synthetic AMPs can effectively prevent and treat infections. Furthermore, during sepsis, microbial products can trigger harmful inflammation through pattern recognition receptors (PRRs). AMPs may counteract this inflammation, but further research is needed to understand their role in mitigating inflammation during infections [149].

In the era of antibiotic resistance, AMPs, whether used individually, in combinations, or as agents inducing their expression (e.g., TLR agonists), may serve as alternatives to antibiotics. Their broad antibacterial mechanism of action makes the development of bacterial resistance less likely [149].

While some studies suggest the potential of lactoferrin and other AMPs in preventing LOS in VLBW infants, there is a lack of neonatal clinical trials for other AMPs. Future research should focus on conducting clinical trials in neonates to explore the efficacy and safety of AMP-based therapies. Innovative approaches like inhaled TLR ligands and synthetic TLR agonists (e.g., PUL-042) may stimulate AMP production and reduce pneumonia in at-risk infants [209,210]. Research should assess their effectiveness in neonates, particularly ventilated premature or VLBW infants [211].

In summary, research on AMPs in infants, especially in high-risk populations, shows promise for preventing and treating infections. Future investigations should address gaps in understanding AMPs’ mechanisms, clinical efficacy, and their potential role in combating antibiotic resistance.

## Figures and Tables

**Table 1:** Microbiome, antimicrobial peptides, and possible interactions. BPI: bactericidal permeability-increasing protein; BPD: bronchopulmonary dysplasia; GI: gastrointestinal; HAMLET: human α-lactalbumin made lethal to tumor cells; human hBD = human beta-defensin; HNP: human neutrophil peptide; LF: lactoferrin, MUC: mucin; NEC: necrotizing enterocolitis; SLPI: secretory leukocyte protease inhibitors.

Organ	Predominant Microbiome in Preterm/Term Infants	Most Abundant AMPs and (Potential) Functions	Possible Clinical Consequences of an Altered Microbiome	Possible Interaction of AMPs and Microbiome/Possible Future Therapeutic Options:	References
**Skin**	**Preterm infants**: *Staphylococcus* spp., *Corynebacterium* spp., *E. coli*, *Enterobacter* spp., *Prevotella* spp., *Lactobacillus*, *Streptococcus* spp. **Term infants**: higher abundance of Proteobacteria, Bacteroidetes, lower abundance of *Staphylococcus* spp.	**Defensins**: mainly active against *S. aureus* **cathelicidin LL-37** **psoriasin** RNase 7: prevents *S. aureus* colonization **dermcidin** **adrenomedullin** All exhibit broad immune protection, effector molecules in regulation and interaction with immune system No differences in AMP levels between preterm and term infants, increased levels in infants with history of maternal chorioamnionitis	Atopic dermatitis Allergies Sepsis risk	*S. epidermis* induces expression of AMPs, e.g., **hBD-2** and **hBD-3** in keratinocytes *S. epidermis* activates IL-1ß AMPs promote stabilization of the skin microbiome	[69,83,90,101,102,120,121,122,123,124,125,126,127,128,129,130,131]
**Lungs**	**Preterm infants**: dominated by *Staphylococcus* spp., *Ureaplasma* spp. and Proteobacteria **Term infants**: *Streptococcus*, *Neisseria*, *Prevotella*, *Prophyromonas*, *Veillonella* and *Fusobacterium*	**α- and β- defensins:** **hBD-2**: predominant, increases with gestational age **cathelicidin LL 37**: potential role in tissue repair **antiproteases elafin and SLPI**: may play a role in preventing ventilator-induced lung injury	Respiratory infection Wheezing Allergic sensitization BPD	Lower **hBD-2** in preterm infants→ possible infections with e.g., *H. influenzae* Lower **SLPI** in mechanically ventilated preterm neonates → associated with ventilator-induced lung injury AMPs modulate lung injury by altering the intestinal microbiota	[16,70,87,92,93,94,95,102,132,133,134,135,136,137,138,139,140,141]
**GI Tract**	**Preterm infants**: *Bifidobacteria* spp., *Bacteroides* spp., *Staphylococcus* spp., *Enterococcus* spp. **Term infants**: high abundance of *Escherichia*, *Klebsiella*	**α- defensins (HNP):** maintain a balanced microbiome From breast milk: **lactoferrin (LF):** inhibits bacterial growth, impairs virulence **lysozyme, LL-37**, **MUC1, MUC4, ß-casein:** prevention of adhesion of pathogens to intestinal lumen **HAMLET:** targets and boosts antibiotic effectiveness **β- defensins**	NEC Infection/sepsis Food allergy, asthma Inflammatory, metabolic, neurologic, cardiovascular and gastrointestinal diseases Impaired outcome at 2 years	Lower **α defensins** → dysbiosis and inflammatory (bowel) diseases Lower **β- defensins** → found in infants with NEC Reduced intestinal AMP expression → in patients with dysbiosis or chronic inflammatory bowel disease **Lactoferrin**: supplementation reduced risk of neonatal sepsis and diarrheal illness	[2,81,84,85,95,96,105,106,142,143,144,145,146,147,148,149,150,151,152,153,154,155,156,157,158]
**Blood**	-	**Defensins** **LL-37** **BPI:** higher levels in patients with bloodstream infection Preterm infants: generally decreased AMP concentrations Neonates have lower levels of AMP compared to adults	-	Preterm infants: decreased concentrations of AMPs → potentially reduced immune protection Higher levels of LF, BPI, alfa-defensins HNP-1, HNP-2 und HNP-3 in newborn cord blood in case of amniotic infection	[70,71,72,73,159,160,161,162]

## Abbreviations

AMPs	antimicrobial peptides
BPI	bactericidal permeability-increasing protein
BPD	bronchopulmonary dysplasia
CS	Caesarean section
hBD	human beta-defensin
HNP	human neutrophil peptide
LF	lactoferrin
LOS	late-onset sepsis
LPS	lipopolysaccharide
MUC	mucin
MRPs	Microbiota-regulation peptides/proteins
NEC	necrotizing enterocolitis
PC	paneth cell
*S. epidermidis*	*Staphylococcus epidermidis*
SLPI	secretory leukocyte protease inhibitors
spp	species
TLR	toll-like receptor
VLBW	very low birth weight
